# Hemolymphangioma of the lower extremities in children: two case reports

**DOI:** 10.1186/1749-799X-5-56

**Published:** 2010-08-12

**Authors:** Ilias Kosmidis, Maria Vlachou, Anastasios Koutroufinis, Konstantinos Filiopoulos

**Affiliations:** 1 Orthopaedic Clinic, Penteli's Children Hospital, Athens, Greece; 2 Mitera" General Maternity Hospital, Athens, Greece

## Abstract

**Background and purpose:**

Hemo-lymphangiomas are rare benign tumors that arise from congenital malformation of the vascular system. They are usually diagnosed at birth or early in childhood. The management of hemo-lymphangiomas in children remains challenging because complete resection is often difficult to be achieved and recurrences are common.

**Methods:**

We present the case of two children with a mass on their left tibia. Imaging modalities, plain radiograph, Ultrasonography and Magnetic Resonance were used to investigate the nature of the mass, the anatomical relationship to the neighboring tissues and help planning the surgical resection. The dominant diagnosis was hemo-lymphangioma. Both lesions increased in size in a short period of follow-up thus we decided to proceed to surgical excision.

The diagnosis of hemo-lymphangioma was confirmed by histological examination of the surgical specimen.

Post-operatively, seroma was formed to the first patient, managed by placing a drainage and immobilizing the limb on a splint.

The second patient experienced no complications post-operatively.

After 12 months of follow-up both patients had no complications or recurrence.

**Conclusions:**

Very few cases of hemo-lymphangiomas of the extremities have been reported in the literature. Those tumors can grow slowly and remain asymptomatic for a long period of time or may become aggressive and enlarge rapidly, without invasive ability though.

Radical resection is the choice of treatment offering the lowest recurrence rates. Other therapeutic methods are: aspiration and drainage, cryotherapy, injection of sclerotic agents and radiotherapy; although none of those offers better results that the surgical excision.

## Introduction

Lymphangiomas are a heterogeneous group of vascular malformations of the lymphatic channels composed of cystically dilated lymphatics. According to Landing and Farber [1] , those benign malformations, are classified in four categories: capillary lymphangioma, cavernous lymphangioma, cystic lymphangioma (hygroma) and hemo-lymphangioma (combination of hemangioma and lymphangioma).

The latter, congenital malformation, can remain asymptomatic for a long period of time. On the other hand, it may grow rapidly, surrounding or infiltrating the neighboring tissues or other major structures, thus making the excision a real challenge for the physician [2,3] .

Hemo-lymphangiomas are in most cases detected at birth or early in a child's life, usually before the age of two years. Alternatively, with the introduction of the prenatal ultrasound, the diagnosis can be placed in the uterus [4] .

This essay is a case report of two children with hemo-lymphangiomas of the lower extremities and its purpose is to define the most effective therapeutic approach of those lesions. Written parental permission was obtained to allow the use of confidential information held in the hospital's records, as Institutional Review Board (IRB) does not exist in our country.

## Case 1

**Figure 1:**
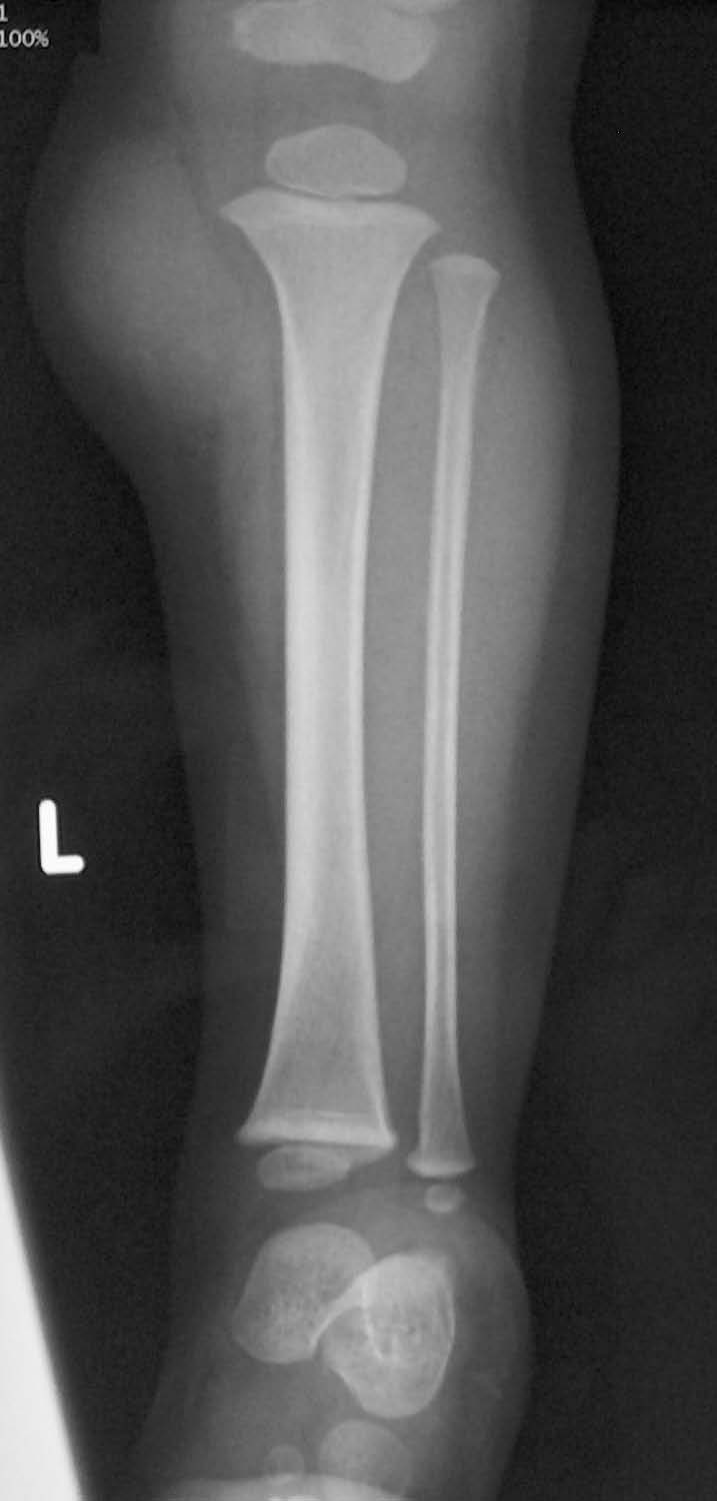
**Case 1- Plain radiograph of the left tibia, demonstrating the mass in the inner-upper part**.

**Figure 2:**
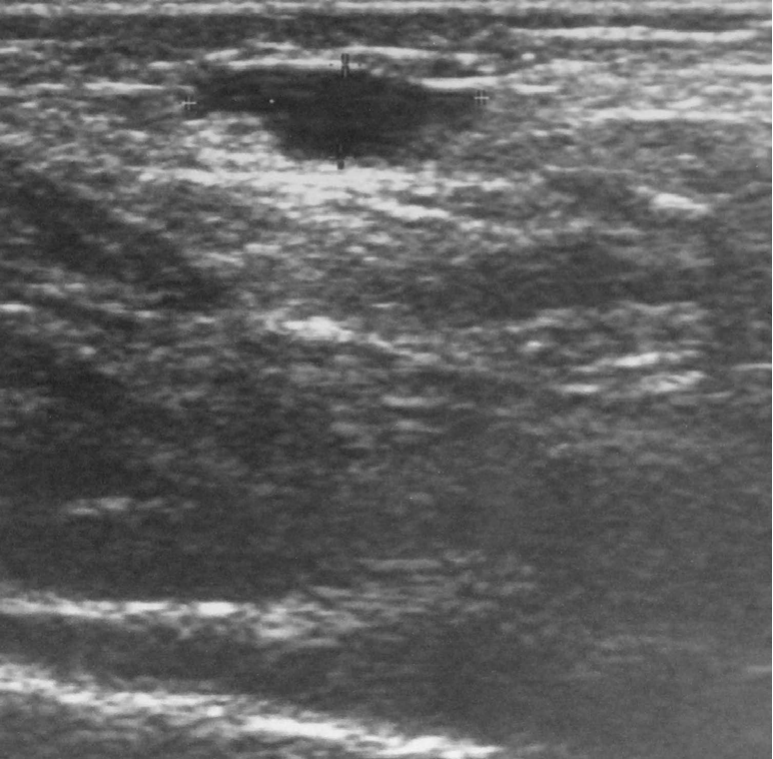
**Case 1- In a six month follow-up, pre-operative Ultrasound measures the tumor 23 mm × 6 mm**.

A two month old female infant presented to the orthopedic examination room with a palpated mass on the anteromedial side of the proximal left tibia. On the physical examination the lesion was found to be oval in shape, soft, compressible and painless. Anteroposterior and lateral plain radiographs demonstrated swelling of the soft tissue at this part of the tibia without signs of bony erosion (Fig. 1). The ultrasound (Doppler sonography) detected a cystic lesion with blood flow, measured 12 mm × 4 mm, while the integrity of the bone cortex was confirmed. The most possible diagnosis was hemo-lymphangioma. In the following six months, despite the fact that the size of the mass increased, the patient remained asymptomatic. A new ultrasound was performed (Fig. 2) and measured the mass 23 mm × 6 mm; the origin remained cystic. In the yearly follow-up the lesion's size increased to 44 mm × 37 mm.

**Figure 3:**
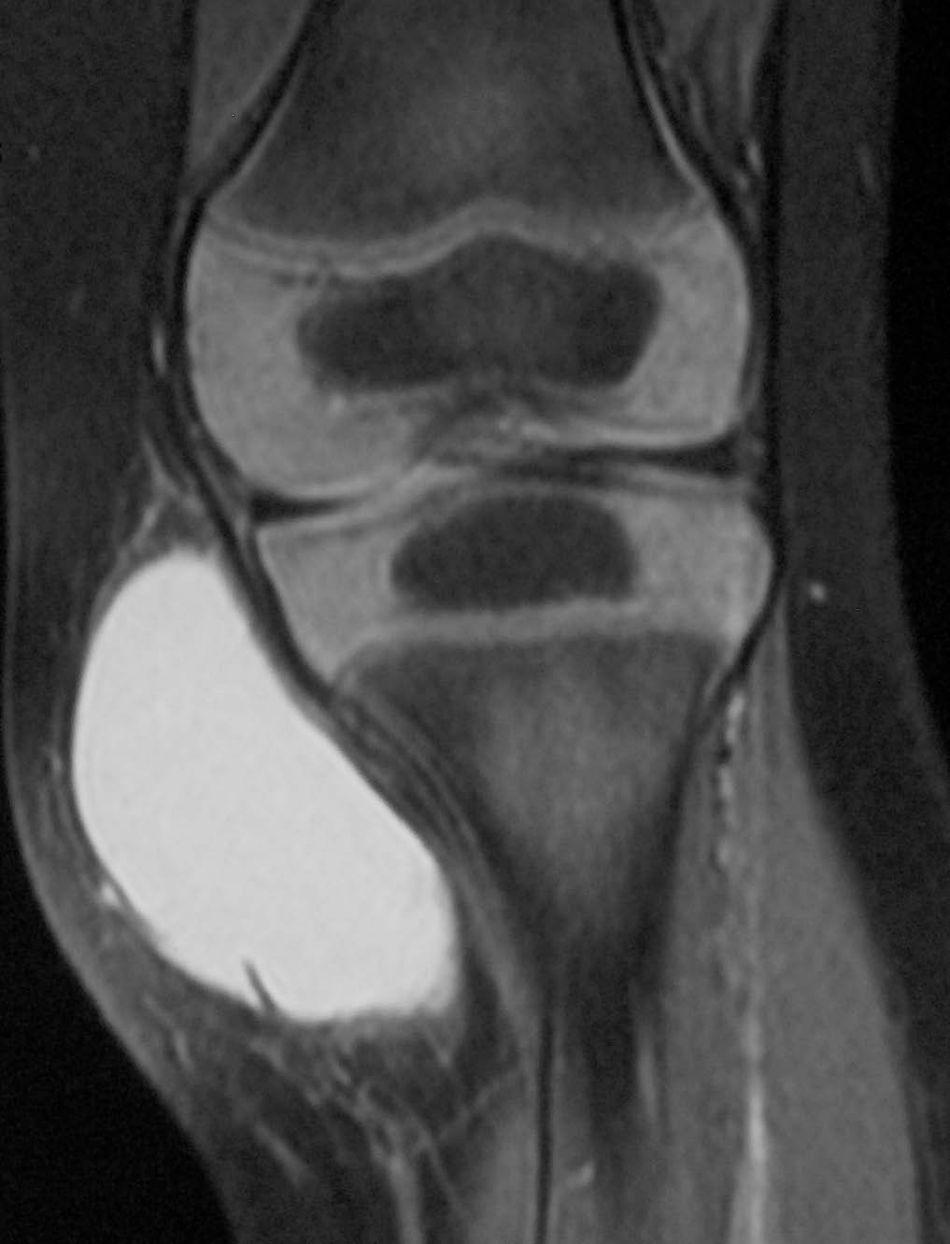
**Case 1- MRI defines the margins of the tumor**.

Due to the continuous augmentation of the mass, surgical excision was decided. An MRI (Fig. 3) was performed, pre-operatively, in order to establish the extent of the tumor and define the relationship to the surrounding structures. A well-defined extra-articular cystic malformation with a maximum diameter of 45 mm was viewed, located on the anteromedial side of the left tibia. The lesion had fine adhesions to the surrounding tissues and the cortex of the bone was found intact.

**Figure 4:**
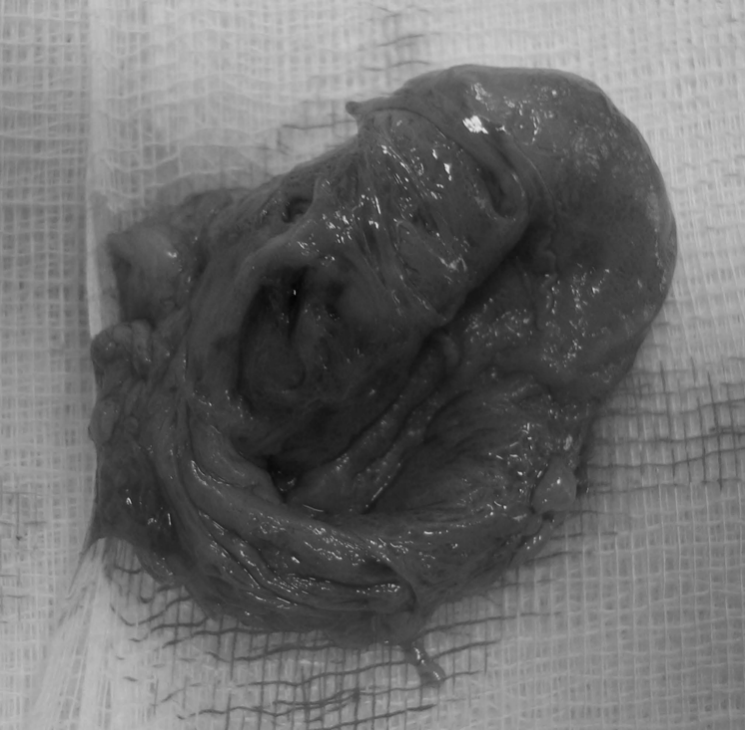
**Case 1- Perioperative aspect of the mass**.

En bloc resection was performed releasing the specimen from the adhesions to the subcutaneous tissues and the medial head of the gastrocnemious. Macroscopically, it was found oval in shape with harsh features and spotty surface (Fig. 4).

**Figure 5:**
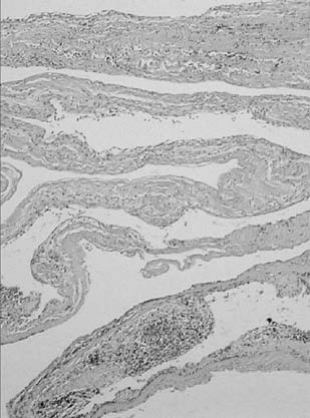
**Histological examination of the mass**. Collapsed and infolded cyst wall.

**Figure 6:**
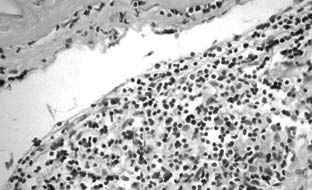
**Histological examination**. Cyst wall structure showing loose connective tissue stroma lined by flat endothelial cells, beneath which is obvious a lymphocytic infiltration.

Histological examination described the resected tumor as a fibro-lipomatous mass containing dense fibrous conjunctive tissue with vascular areas of lymphatic cells and vessels filled in with red blood cells (Fig. 5 &6). The definitive histological diagnosis was hemo-lymphangioma.

Ten days post-operatively the trauma area was swelling; the seroma formed was aspirated and gave 20 ml of sterile, bloody fluid.

A week later, the swelling recurred; surgical exploration and lavage was performed, while a drainage was placed. Intravenous Netilmicin and Cefamandile Nafate were administered for a total period of ten days, whereas the leg was immobilized on an above knee splint. The swelling was progressively decreased. A week after, the trauma area was examined by the ultrasound and 1,58 ml's of fluid were demonstrated. A full plaster was placed with the knee joint flexed in 90° for three weeks. At the end of that period of time the volume of the fluid was less that 1 ml. After two months of follow-up by clinical and Ultrasonography examination, the quantity minimized to zero, while in the yearly follow-up no recurrence was observed.

## Case 2

**Figure 7:**
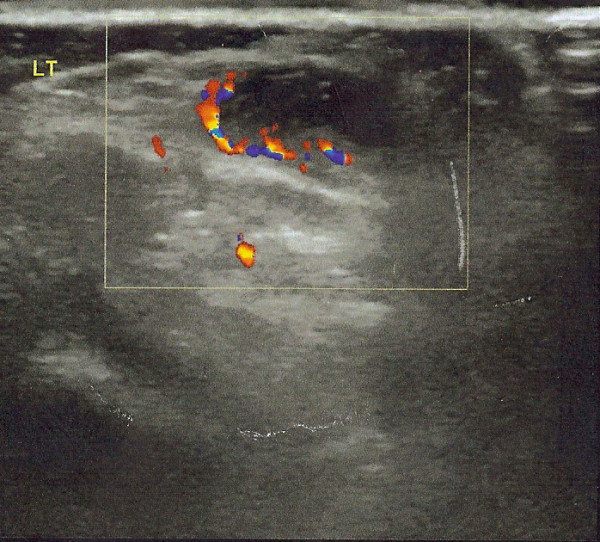
**Case 2- A Doppler Sonography demonstrating the blood flow in the mass**.

The second case regards a 5 year-old male patient, with a palpable, painless mass on the anterior side of the proximal left tibia, located 2 cm below the tibial tubercle. The tumor, within a two month follow-up, was doubled in size; although it remained asymptomatic. A soft tissue swelling and intact bone cortex were demonstrated by the plain radiographs (anteroposterior and lateral views). The ultrasound performed depicted a cystic mass with moderate vascularization, measured 21 mm × 18 mm × 5 mm (Fig. 7).

The treatment decided was en bloc resection of the tumor, followed by rigorous cauterization of the blood and lymphatic vessels; a drainage was placed.

Microscopically, the tumor examined, was described as a soft tissue cystic mass consisted of lymphatic and blood vessels; the stroma was infiltrated by lymphocytes. The histological diagnosis was hemo-lymphangioma.

Cephalosporin was administered, in 3 doses totally, pre and post-operatively, while the limb was immobilized on a splint with the knee joint flexed in 30° for a total period of three weeks. In the trauma area no seroma was formed thereby, we removed the drainage.

**Figure 8:**
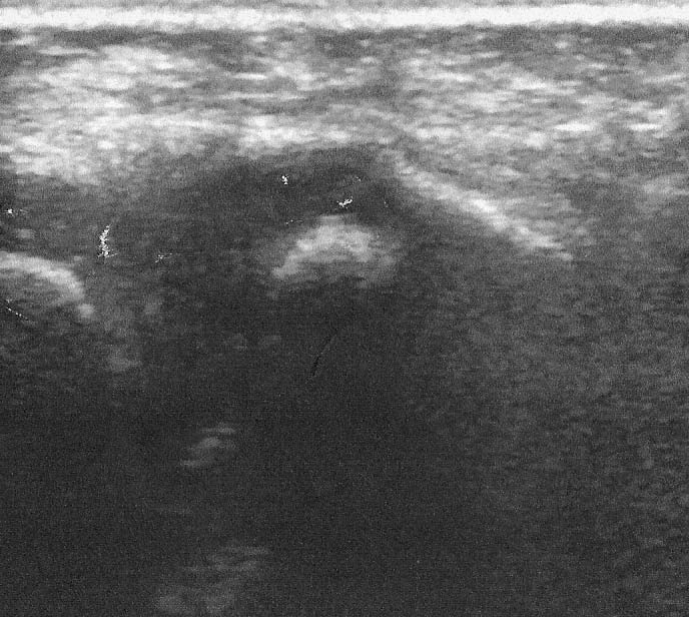
**Case 2- The Ultrasound verifies the absence of recurrence **.

At the yearly follow-up no recurrence was observed, verified by the use of Ultrasonography (Fig. 8).

## Discussion

Hemo-lymphangiomas are rare benign tumors that appear to arise from congenital malformation of the vascular system. The formation of that tumor may be explained by obstruction of the venolymphatic communication, between dysembrioplastic vascular tissue and the systemic circulation [5] . Hemo-lymphangiomas are mostly presented as cystic or cavernous lesions.

Very few cases of hemo-lymphangiomas of the extremities have been reported in the literature. A retrospective study of one hundred and eighty-six (186) patients' presenting one hundred and ninety-one (191) hemangiomas was published; their anatomical location was: 48% in the head and neck, 42% in the extremities and 10% in internal or visceral locations. Histological examination revealed that only three of them had combined hemo-lymphangiomas [6] . Macroscopically, complete excision gave the best results with lower recurrence rate. On the contrary, aspiration and injection of sclerotic agents gave the highest risk of recurrence. Among the most important risk factors for recurrence are: anatomical location of the lesion, size, complexity and surgical technique. In that study, the average follow-up period was three years, while 95% of the patients completed a sufficient period of twelve months.

The incidence of hemo-lymphangiomas varies from 1,2 to 2,8 per 1000 newborns[7] however, only 6,5% of them are located on the extremities [8,9] ; both sexes get equally affected. The diagnosis in most cases (90%) is placed before the age of two years [2] , while 60% of those are present at the time of birth.

The clinical on set of hemo-lymphangiomas can vary from a slowly growing cyst over a period of years to an aggressive enlarging tumor, without invasive ability. Their size varies due to the anatomical location and relationship to the neighboring tissues. Small tumors are usually superficial, while the large ones are located deeper and have cystic texture. The most common complications are random or traumatic hemorrhage, rupture, infection and lymphorrhya. In the clinical examination they are usually described as soft and compressible masses, loculated in pattern. Histologically, hemo-lymphangiomas consist of dense fibrous tissue that grows in bands between the numerous vascular spaces and invades the subcutaneous fat. Some of those are blood vessels and the others lymphatic.

Imaging modalities, Ultrasonography, Computed Tomography and Magnetic Resonance, are useful in confirming the diagnosis, describing the margins of the mass and planning the surgical strategy [10] . The ultrasound, that is a low cost modality, may demonstrate the solid or cystic nature of the lesion, therefore should be ordered in a routine base; the extension and the relationship of the tumor to the surrounding tissues is rather depicted by the MRI [11]. The differential diagnosis includes hemangioma, lipoma, teratoma, congenital lymphedema, dermoid cyst and neurofibroma. Needle aspiration or biopsy can also be useful in the differentiating hemo-lymphangioma to other fluid-filled masses.

As far as it concerns the therapeutic approach of localized hemo-lymphangiomas, the treatment of choice is complete surgical excision, which also presents the lowest recurrence rate. An important issue regards the time of surgery. Some authors express the aspect that the mass should be excised promptly after being diagnosed, while others prefer to wait and examine the size regularly. In the latter case, the risk of infection and hemorrhage remains, while complete surgical eradication may become more difficult. Other techniques available are: aspiration and drainage, cryotherapy, injection of sclerotic agents and radiotherapy (by radium, roentgen ray or radon seed), but none of them produced acceptable results. Radiotherapy is used when surgical excision is not feasible; the radio-sensitivity of hemo-lymphangiomas is not well understood, however in the past they were considered to be radio-resistant. In children, radiotherapy may lead to tumor retardation or to malignant transformation [12]. When surgical eradication fails and the mass recurs, conservative treatment methods may be applied. The recurrence rates vary depending on the complexity of the mass, the anatomical location and the adequacy of the excision. However, lesions that have been completely excised, present 10-27% recurrence, while those being partially resected may recur in 50-100%.

An important issue remains the continuous lymphorrhya during the instant post-operative period; attentive en bloc excision of the tumor, followed by rigorous cauterization of the blood and lymphatic vessels may reduce or eliminate the risk of recurrence. Other auxiliary measures are the application of a bandage under pressure, the prolonged drainage and immobilization of the extremity.

## Conclusions

In the treatment of hemo-lymphangioma, surgical excision appears to be the best choice of treatment, especially when the tumor increases in size, creating pressure to the surrounding tissues. Eradicate attentive excision offers the minimum risk of recurrence.

## Competing interests

The authors declare that they have no competing interests.

## Authors' contributions

IK, who is the corresponding author, was the surgeon of the first patient, gathered the articles used as references and compiled the manuscript. M.V was the surgeon of the second patient. A.K helped with the editing of the manuscript. KF, as the director of the orthopaedic department, guided us and helped in finalizing the manuscript.

All authors have read and approved the final manuscript

## Consent

Written informed consent was obtained from the patients for publication of this case report and any accompanying images. A copy of the written consent is available for review by the Editor-in-Chief of this journal.
